# Ozone Exerts Cytoprotective and Anti-Inflammatory Effects in Cardiomyocytes and Skin Fibroblasts after Incubation with Doxorubicin

**DOI:** 10.1155/2019/2169103

**Published:** 2019-11-18

**Authors:** V. Simonetti, V. Quagliariello, M. Franzini, R. V. Iaffaioli, N. Maurea, L. Valdenassi

**Affiliations:** ^1^“Kaos” ONLUS Foundation, Turin, Italy; ^2^Oxygen-Ozone Therapy Scientific Society (SIOOT), Gorle, Italy; ^3^Division of Cardiology, Istituto Nazionale Tumori-IRCCS-Fondazione G. Pascale, Napoli, Italy; ^4^University of Pavia, Pavia, Italy; ^5^ASMO (Association of Multidisciplinary Study in Oncology) and Mediterranean Diet, Piazza Nicola Amore, Napoli, Italy

## Abstract

**Introduction:**

Skin reactions and cardiotoxicity are one of the most common side effects of doxorubicin in cancer patients. The main mechanisms based on the etiopathogenesis of these reactions are mediated by the overproduction of proinflammatory cytokines, metalloproteases, and the disruption of mitochondrial homeostasis. Ozone therapy demonstrated anti-inflammatory effects in several preclinical and clinical studies. The aim of this research is based on the evaluation of cardioprotective and dermatoprotective effects of ozone during incubation with doxorubicin, giving preliminary evidences for further studies in the field of cardio-oncology.

**Methods:**

Human skin fibroblast cells and human fetal cardiomyocytes were exposed to doxorubicin at subclinical concentration (100 nM) alone or combined with ozone concentrated from 10 up to 50 *μ*g/mL. Cell viability and multiple anti-inflammatory studies were performed in both cell lines, with particular attention on the quantification of interleukins, leukotriene B4, NF-*κ*B, and Nrf2 expressions during treatments.

**Results:**

Ozone decreased significantly the cytotoxicity of doxorubicin in skin fibroblasts and cardiomyocytes after 24 h of incubation. The best cytoprotective effect of ozone was reached to 30 *μ*g/mL with a plateau phase at higher concentration. Ozone also demonstrated anti-inflammatory effects decreasing significantly the interleukins and proinflammatory mediators in both cells.

**Conclusion:**

Ozone exerts cardioprotective and dermatoprotective effects during incubation with doxorubicin, and the involved mechanisms are mediated by its anti-inflammatory effects. The overall picture described herein is a pilot study for preclinical studies in oncology.

## 1. Introduction

Anthracyclines and target-based drugs like anti-ERB2 and VEGF antibodies are conventionally used in cancer patients both in combination or in monotherapy. However, their clinical use is limited by a wide spectrum of reversible and irreversible side effects like cardiovascular diseases as well as skin inflammation [1, 2]. For example, anthracycline-induced cardiotoxicity is a common side effect seen in cancer patients especially in those with many cardiovascular risk factors such as metabolic syndrome, previous stroke, and other comorbidities like diabetes [2]. Moreover, anthracyclines induce apoptosis and necrosis of hepatic, renal, and cutaneous cells, modifying dramatically the quality of life of cancer patients [3]. The main mechanisms of these side effects are based on the induction of free radicals and the overproduction of proinflammatory markers, strictly associated to proapoptotic signals [4, 5]. Recently, it was demonstrated that an important mediator of doxorubicin-induced cardiotoxicity is the overproduction of interleukin-1 in cardiac cells that is correlated to the increases of hs-C-reactive protein (hs-CRP), defined as the key inductor of inflammation and a common cardiovascular risk factor [6, 7]. The cardiac toxicity of anthracyclines is also mediated by the alteration of calcium homeostasis, leading to the activation of intracellular proteases involved in cell necrosis [7]. Ozone therapy is an alternative medicine of great clinical interest also in the field of oncology especially for the management of dermatological and cartilaginous side effects [8–10]. Ozone therapy is based on the use of ozone, a highly reactive unstable gas, at different concentrations, generally from 10 to 50 *μ*g/mL, in different organs. The ozone therapist uses conventionally ozone as a tool to disinfect hospital instruments and tissues, as well as to reduce the general pain and inflammation [11]. From a biochemical point of view, it was recently demonstrated that ozone has a pro-oxygenating effects and stimulates the repair of damaged tissues through the nuclear factor-erythroid 2-related factor 2(Nrf2), a key transcription factor involved in the metabolic homeostasis of human cells. Considering that Nrf2 is of crucial importance in cancer cell survival as well as in the regeneration of skin, the exploration of ozone-related multiple effects in cancer patients could be of great interests for clinicians [12, 13]. Our research group demonstrated that ozone, exposed to colon cancer [14] and melanoma [15] cells, increases the anticancer action of conventional anticancer drugs like 5-fluorouracile and exerts anti-inflammatory effects. Furthermore, we have shown that ozone is able to improve the tumor microenvironment by reducing the production of cytokines involved in cancer cell survival and chemoresistance; these effects could be of great interest in oncology considering that the tumor microenvironment has a key role in the management of the chemoresistance phenomena to many drugs like the anthracyclines as well as to immunotherapies. The aim of this study is based on demonstrating, for the first time, the cardioprotective and dermatoprotective effects of ozone, at different concentrations, during exposure of cells to doxorubicin, through the analysis of mitochondrial homeostasis, cellular inflammatory state, and the expression of the Nrf2.

## 2. Materials and Methods

### 2.1. Dermato- and Cardioprotection Studies: Mitochondrial Dehydrogenase Activity Assay

The cytoprotective effects of ozone were evaluated on the human skin fibroblast cell line (CRL1474) (purchased from the American Type Culture Collection, USA) and on HL-1 atrial muscle cell line (SCC065, purchased from Sigma Aldrich, Milan, Italy). To this aim, we quantified the cellular mitochondrial dehydrogenase activity by the [3-(4,5-dimethylthiazol-2-yl)-2,5-diphenyltetrazolium bromide] method according to the instructions provided by Dojindo Molecular Technologies Inc. Cardiomyocytes were cultured in a Claycomb medium (Sigma Aldrich, Milan, Italy) containing 10% fetal bovine serum, 100 U/mL penicillin/streptomycin, 0.1 mM norepinephrine, and 2 mM L-glutamine and maintained at 37°C in 5% CO_2_ in a humidified atmosphere. Skin fibroblasts were grown in Dulbecco's modified Eagle's medium (DMEM; Gibco; Thermo Fisher Scientific, Inc) with 10% fetal bovine serum and 1% penicillin and streptomycin solution and incubated at 37°C in a humidified atmosphere containing 5% CO_2_. After one day of appropriate growth, cells were incubated with ozone, from 10 to 50 *μ*g/mL, produced with Multiossigen machinery, type Medical 99 IR, following the same method described in literature [13, 14], alone or in combination with doxorubicin (ranged from 0.01 to 50 *μ*M) for 24 h. After the incubation period, both cells were washed slowly in PBS at pH 7.4 and incubated with 100 *μ*l of a MTT solution for 4 h at standard growth condition, as described in other previous works [16]. The absorbance readings were acquired at 450 nm through a Tecan Infinite M200 plate reader with the associate I-control software. The relative cell viability (%) was calculated by the formula [A] test/[A] control × 100, where “[A] test” is the absorbance of the test sample and “[A] control” is the absorbance of the control cells incubated solely within the culture medium. The cytotoxicity was then normalized by the amount of total protein content quantified through the Micro BCA protein assay kit (Pierce).

### 2.2. Determination of Mitochondrial Matrix Potential in Cardiomyocytes and Skin Fibroblasts

MitoProbe™ JC-1 Assay Kit (Thermo Fisher kit) was used for the determination of the mitochondrial matrix potential (MMP) in skin fibroblasts and cardiomyocytes, according to the manufacturer's protocol. Following treatment with doxorubicin at subclinical concentration (100 nM) and ozone at 10, 20, 30, 40, and 50 *μ*g/mL or both in combination, 1 × 10^5^ cells were cultured in 24-well plates and incubated with a solution 5 *μ*g/mL of JC-1 for 20 min at standard growth condition. Cells were subsequently rinsed twice with JC-1 staining buffer, and the fluorescence intensities of mitochondrial JC-1 monomers and aggregates (*λ*ex.514 nm; *λ*em.529 nm/*λ*ex.585 nm; *λ*em.590 nm, respectively) were quantified through a Tecan Infinite M200 plate reader with the associate I-control software. The MMP of fibroblasts and cardiomyocytes was calculated as the red/green fluorescence ratio.

### 2.3. Activation of p65/NF-*κ*B in Cardiomyocytes and Skin Fibroblasts

Fibroblasts and cardiomyocytes were exposed to doxorubicin at 100 nM alone or in combination with ozone at different concentrations for 6 h, and then nuclear extracts were analyzed using the TransAM NF-*κ*B transcription factor assay kit (Active Motif, Carlsbad, CA), according to the manufacturer's recommendations. NF-*κ*B complexes were captured by binding to a consensus 5′-GGGACTTTCC-3′ oligonucleotide immobilized on a 96-well plate. Bound NF-*κ*B was quantified by incubating with anti-p65 primary antibody followed by horseradish peroxidase- (HRP-) conjugated goat anti-rabbit IgG and spectrophotometric detection at a wavelength of 450 nm using a microplate spectrofluorometer (xMark microplate spectrofluorometer, Bio-Rad, Milan, Italy). Data were expressed as the percentage of NF-*κ*B/DNA binding relative to control cells.

### 2.4. Nrf2 Activity in Cardiomyocytes and Skin Fibroblasts

For the evaluation of Nrf2 activity during incubation with doxorubicin (100 nM) and ozone (from 10 to 50 *μ*g/mL), we performed a nuclear extraction in both cell lines, and 10 *μ*g of the extracts were incubated in a 96-well plate that was coated with oligonucleotides containing a consensus binding site for Nrf2. After 1 h of incubation, we washed the plates three times, slowly, with cold PBS and incubated with 100 *μ*l of a 1 : 1000 dilution of polyclonal antibody against Nrf2. After the appropriate washing with cold PBS, we incubated the plates with 100 *μ*l of a 1 : 1000 dilution of horseradish peroxidase-conjugated, anti-secondary antibody at 25°C. Then, the fold expression of Nrf2 was obtained dividing the absorbance of treated cells and untreated (control) cells at a wavelength of 450 nm using a microplate spectrofluorometer (xMark microplate spectrofluorometer, Bio-Rad, Milan, Italy).

### 2.5. Protein Expression of Leukotriene Type B4 in Cardiomyocytes and Skin Fibroblasts

Leukotriene B4 expression in cardiomyocytes and skin fibroblasts was determined through the following method: both cells were incubated with arachidonic acid (10 *μ*M) as the positive control because it is the main precursor of leukotrienes, doxorubicin (100 nM) alone or coincubated with ozone at 10, 20, 30, 40, and 50 *μ*g/mL. During the incubation period, cells were also exposed to 5 *μ*M calcium ionophore A23187, 1.6 mM CaCl_2_ at 37°C for 4 h. Then, we performed the appropriate ELISA method for the quantification of leukotriene type B4, expressed as pg/mL (Cayman Chemical) following the supplier's instructions [17].

### 2.6. Expression of Proinflammatory Cytokines and Metalloproteases in Cardiomyocytes and Skin Fibroblasts

We quantified the expression of proinflammatory cytokines and metalloproteases involved in the skin and cardiac toxicities induced by chemotherapies through the ELISA methods, as described in detail in other works [18, 19]. After the appropriate cell growth and starvation in serum-free medium for 2.5 h, the cells were treated with or without ozone (from 10 up to 50 *μ*g/mL) for 5 h before exposure to doxorubicin always at subclinical concentration (100 nM) for 12 h. As performed for the other experiments, it is crucial to say that we decided to use this concentration of anticancer drug for simulating at cellular level the main clinical exposure of organs (like the heart) to doxorubicin after intravenous administration in humans [20]. After treatments, culture supernatants were collected, centrifuged to pellet any detached cells. Specific ELISAs for the quantification of interleukin-1, interleukin-6, interleukin-8, tumor necrosis alpha, metalloprotease types 9 and 2 were performed according to the manufacturer's instructions. The sensitivity of these methods was less than 10 (pg/mL), and the assay can accurately detect cytokines in the range of 1–32,000  pg/mL.

### 2.7. Statistical Studies

The results reported are the average of three independent experiments ± SD (standard deviation). We performed a one-way analysis of variance (ANOVA) and by a subsequent Tukey's multiple comparison test in SigmaPlot Software for the determination of the difference between the groups. For statistical analysis of all data, *p* < 0.05 is considered as statistically significant.

## 3. Results

### 3.1. Dermato- and Cardioprotection Studies

**Figure 1:**
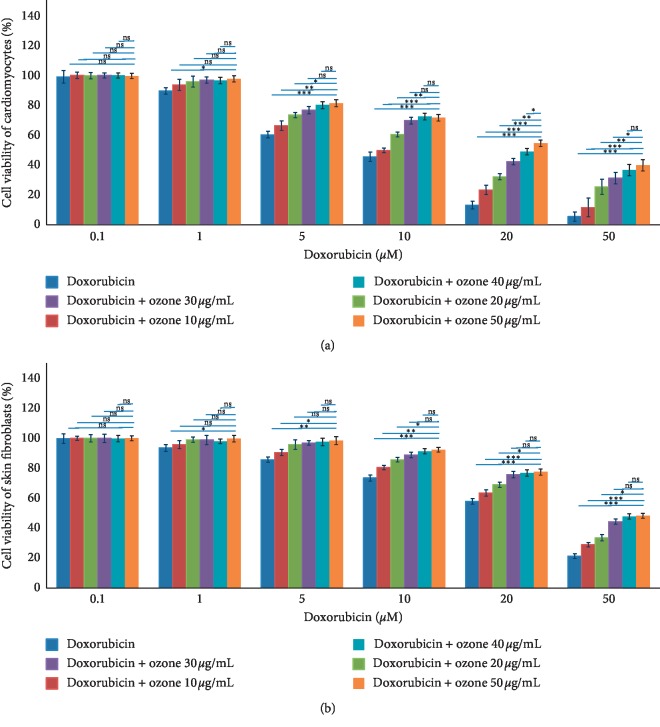
Cardiomyocyte (a) and skin fibroblast (b) cell viability (±SEM) performed by the modified MTT method after incubation for 24 h with doxorubicin and ozone-doxorubicin coexposure. ^*∗*^*p* < 0.001; ^*∗∗*^*p* < 0.05; ns: not significant.

As shown in Figure 1, doxorubicin has cytotoxic effects in both cell lines. Specifically, in cardiomyocytes, the IC50 at 24 h of incubation is around 10 *μ*M, in agreement with our previous research [7] in cardiomyoblasts. Coincubation with ozone leads to an enhanced viability of cardiomyocytes with an IC50 of 25 *μ*M at the higher ozone concentration (50 *μ*g/mL). Coincubation of cardiomyocytes with doxorubicin 20 *μ*M and ozone at 10, 20, 30, 40, and 50 *μ*g/mL increases their viability 1.8, 2.5, 3.3, 3.6, and 4.2 times, respectively, compared to only doxorubicin-treated cells (*p* < 0.05 for all). Coincubation of cardiomyocytes with doxorubicin 50 *μ*M and ozone at 10, 20, 30, 40, and 50 *μ*g/mL increases their viability 2.1, 4.5, 5.5, 6.4, and 7.3 times, respectively, compared to only doxorubicin-treated cells (*p* < 0.05 for all). A similar but lower significative behaviour was seen in skin fibroblasts with an overall cytoprotective effects of 10–15% of ozone at higher concentration coincubated with doxorubicin. As an example, coincubation of skin fibroblasts with doxorubicin 50 *μ*M and ozone at 10, 20, 30, 40, and 50 *μ*g/mL increases their viability 1.3, 1.6, 2.1, 2.3, and 2.4 times, respectively, compared to only doxorubicin-treated cells (*p* < 0.05 for all).

### 3.2. Measurement of Mitochondrial Matrix Potential (MMP) in Fibroblasts and Cardiomyocytes during Doxorubicin Exposure

**Figure 2:**
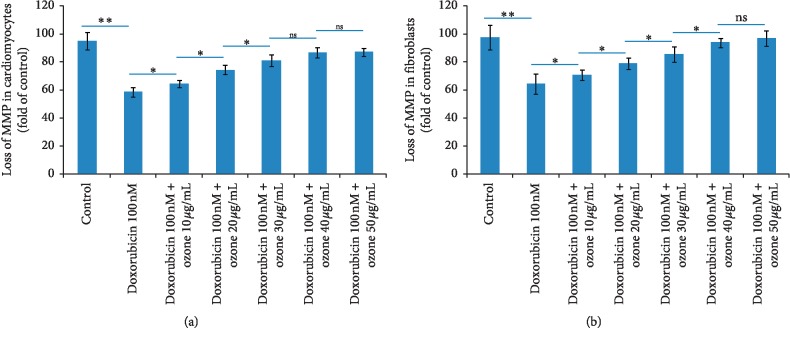
The MMP of cardiomyocytes (a) and skin fibroblasts (b) determined by a JC-1 kit, relative to the control (untreated) cells. Values are presented as the mean ± standard error of the mean (*n* = 3).

To understand the mechanism of ozone-related cytoprotective effects, we investigated the loss of mitochondrial matrix potential after incubation with doxorubicin and ozone-doxorubicin. Following treatment with doxorubicin at subclinical concentration [20], the MMP of cardiomyocytes and skin fibroblasts was significantly decreased of around 39 and 34.6% compared with the untreated cells (*p* < 0.001 for both). Coincubation with ozone leads to an improvement of mitochondrial matrix potential in cardiomyocytes 10.2, 28, 36, 43.2, and 46.1% compared to only doxorubicin-treated cells (*p* < 0.005 for all). A similar behaviour was seen in skin fibroblasts, where the coincubation of ozone with doxorubicin improves the mitochondrial matrix potential in cardiomyocytes 10.2 (not significant), 21.1, 29.4, and 32% compared to only doxorubicin-treated cells (*p* < 0.005 for all) (Figure 2).

### 3.3. NF-*κ*B Transcription Factor Assay

**Figure 3:**
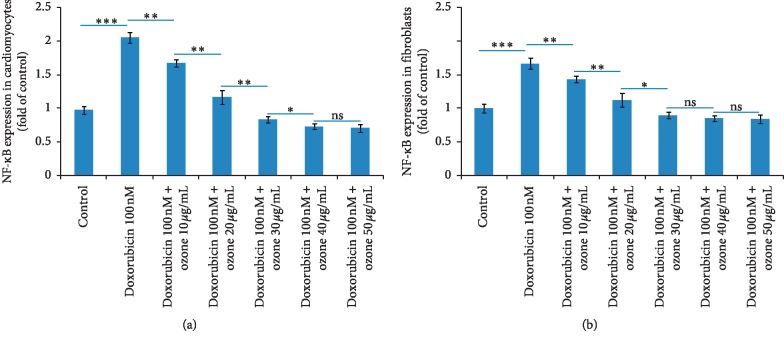
Effects of ozone on NF-*κ*B activation. Human fetal cardiomyocytes (a) and skin fibroblasts (b) were pretreated with various concentrations of ozone (10, 20, 30, 40, and 50 *μ*g/mL) and doxorubicin for 24 h. Nuclear extracts were prepared, and NF-*κ*B activation was measured by enzyme-linked immunosorbent assay. Quantified data are expressed as the mean ± standard error of the mean of three experiments. ^*∗*^*p* < 0.001; ^*∗∗*^*p* < 0.05; ns: not significant.

The activation of the key proinflammatory mediator NF-*κ*B plays a crucial role in cardiovascular diseases, cardiotoxicity, and skin adverse effects of several anticancer drugs. To investigate whether ozone affects NF-*κ*B activation, which is critical for transcriptional activity, the DNA binding activity of NF-*κ*B was analyzed by ELISA. As shown in Figure 3, doxorubicin at subclinical concentration increases of around two times and 1.6 times the expression of p65/NF-*κ*B in cardiomyocytes and skin fibroblasts, respectively (*p* < 0.001 for both) compared to untreated cells. Notably, the coincubation of doxorubicin and ozone leads to a concentration-dependent reduction of p65/NF-*κ*B expression with a plateau phase at 40 and 50 *μ*g/mL. In cardiomyocytes (Figure 3, left), ozone at 10, 20, 30, and 40 *μ*g/mL decreased the p65/NF-*κ*B expression 19.2, 44.1, 60.3, and 65% compared to only doxorubicin-treated cells (*p* < 0.001 for all). In skin fibroblasts (Figure 3, right), ozone at 10, 20, 30, and 40 *μ*g/mL decreased the p65/NF-*κ*B expression 15.2, 33, 47.3, and 50.2% compared to only doxorubicin-treated cells (*p* < 0.001 for all). Increasing ozone concentration over 40 *μ*g/mL did not show more anti-inflammatory activity related to NF-*κ*B activity, indicating a plateau phase.

### 3.4. Leukotriene B4 Expression

**Figure 4:**
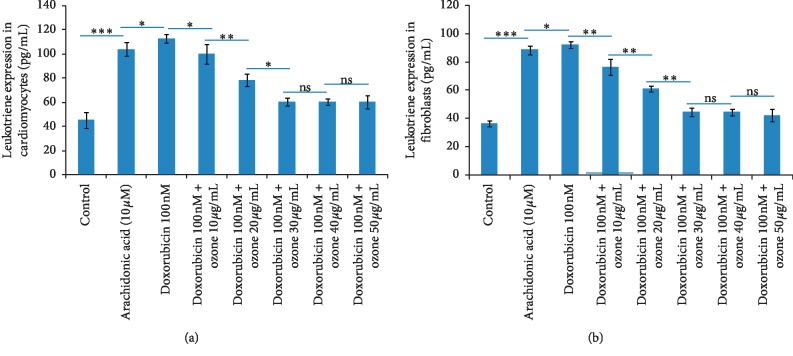
Leukotriene B4 expression (pg/mL) in cardiomyocytes (a) and skin fibroblasts (b) exposed to arachidonic acid (10 *μ*M), doxorubicin (100 nM) alone, or coincubated with ozone at 10, 20, 30, 40, and (50 *μ*g/mL). ^*∗*^*p* < 0.001; ^*∗∗*^*p* < 0.05; ns: not significant.

Leukotrienes are the progenitors of the inflammation related to the activation of prostaglandins [21]. The inflammation induced by leukotrienes is considered of interest in cardiology as well as in oncology considering its activation of oncogenic and cardiotoxic interleukins and NF-kB [22]. Arachidonic acid stimulates the production of leukotriene B4 2.3 times in cardiomyocytes and skin fibroblasts compared to the untreated ones (*p* < 0.001 for both). Incubation with doxorubicin increases around 2.8 times the leukotriene B4 production compared to untreated cells (*p* < 0.001 for both). Notably, in cardiomyocytes (Figure 4, left) ozone at 10, 20, and 30 *μ*g/mL decreased the leukotriene B4 expression 12.2, 31, and 45.4% compared to only doxorubicin-treated cells (*p* < 0.001 for all). In skin fibroblasts (Figure 4, right), ozone at 10, 20, and 30 *μ*g/mL decreased the leukotriene B4 expression of 17, 33.2, and 49.3% compared to only doxorubicin-treated cells (*p* < 0.001 for all). Also in this case, ozone at 40 and 50 *μ*g/mL did not show any greater anti-inflammatory effects both in cardiomyocytes and skin fibroblasts.

### 3.5. Activity of Nrf2

**Figure 5:**
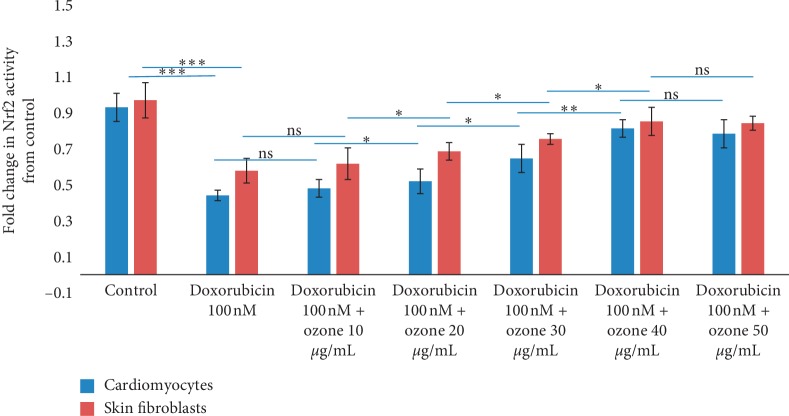
Nrf2 binding activity in cardiomyocytes and skin fibroblasts by doxorubicin (100 nM) and ozone-doxorubicin treatments. Values are expressed as fold change in Nrf2 activity from untreated (control) cells.

Nuclear factor erythroid 2-related factor 2 (Nrf2) is a transcription factor that plays a significant role in regulating the expression of antioxidant and cytoprotective enzymes in response to oxidative stress [23]. Herein, we tested the effects of ozone in the activation of Nrf2 during incubation with doxorubicin; cells exposed to chemotherapy decrease significantly of more than 50% the expression of Nrf2 (Figure 5), and this behaviour should be strictly related to the activation of oxidative stress and inflammation of doxorubicin [1]. Interestingly, in cardiomyocytes (Figure 5, left), ozone at 10, 20, and 30 *μ*g/mL increased the Nrf2 expression 1.2, 1.4, and 1.5 times, respectively, compared to only doxorubicin-treated cells (*p* < 0.001 for all). In skin fibroblasts, we have seen the same behaviour (Figure 5, right), confirming the biochemical effect of ozone described in literature [24, 25].

### 3.6. Anti-Inflammatory Studies

**Figure 6:**
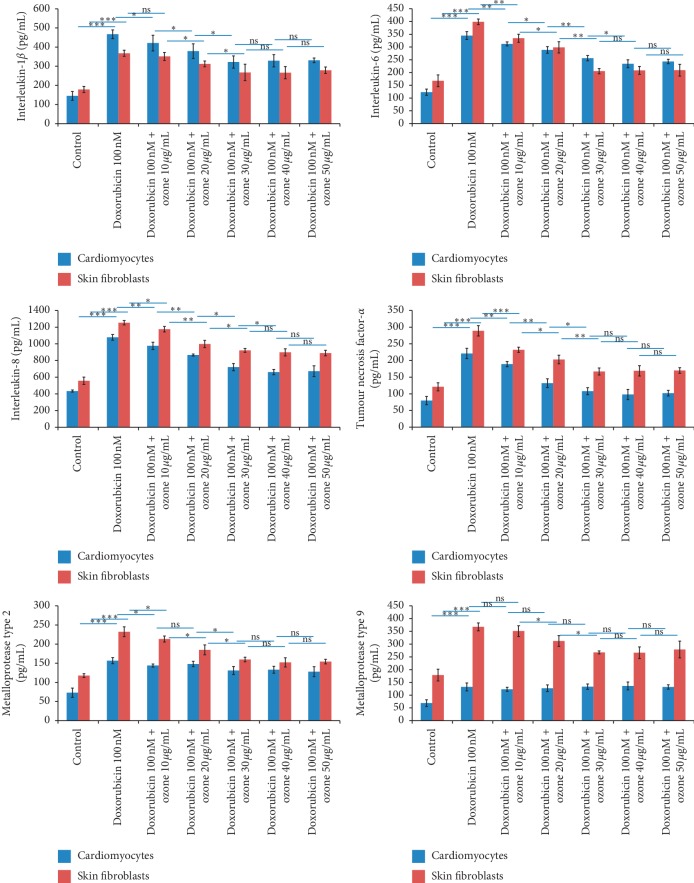
Anti-inflammatory properties of ozone at different concentrations on IL-1, IL-8, IL-6, TNF-*α*, MMP-2, and MMP-9 production by cardiomyocytes and skin fibroblasts (at a density of 1.2 × 10^5^ cells/well). Cells were treated with or without ozone (from 10 up to 50 *μ*g/mL) for 5 h before exposure to doxorubicin at subclinical concentration (100 nM) for 12 h ^*∗*^*p* < 0.001; ^*∗∗*^*p* < 0.05; ns: not significant.

We previously demonstrated anti-inflammatory and anticancer properties of ozone in the cellular model of colon [14] and melanoma cancer cells [15]. Considering our previous experience, we evaluated, for the first time, the putative protective effects of ozone at different concentrations against doxorubicin cytotoxicities. Proinflammatory effects of doxorubicin in cardiomyocytes are well described in our published works [7]. Herein, we demonstrated strong proinflammatory effects of doxorubicin in skin fibroblasts and cardiomyocytes (Figure 6). Notably, pretreatment with ozone decreased significantly the level of all molecules analyzed in a concentration-dependent manner (Figure 6). Specifically, in cardiomyocytes, doxorubicin at 100 nM increased the production of IL-1, IL-8, IL-6, TNF-*α*, MMP-2, and MMP-9 3.2, 2.5, 3, 2.8, 2.3, and 2 times, respectively, compared to untreated cells. Ozone at 10, 20, and 30 *μ*g/mL decreased 10–13, 21–25, and 23–28% the production of all analyzed cytokines and growth factors compared to only doxorubicin-exposed cells (*p* < 0.05 for all). Increasing ozone concentrations seems to not decrease significantly the production of these cytokines indicating a plateau phase, corroborating biological data described in previous paragraphs. Moreover, in skin fibroblasts, doxorubicin at 100 nM increased the production of IL-1, IL-8, IL-6, TNF-*α*, MMP-2, MMP-9, 2, 2.3, 2.2, 2.4, 2, and 2.1 times, respectively, compared to untreated cells. Ozone at 10, 20, and 30 *μ*g/mL decreased 8–12, 18–21, and 20–22% the production of these cytokines and growth factors compared to only doxorubicin-exposed cells (*p* < 0.05 for all). Also in this case, greater doses of ozone do not appear to have a greater anti-inflammatory effect thus having a pattern similar to that observed in cardiomyocytes (Figure 6).

## 4. Discussion

Doxorubicin is a cytotoxic drug used from several years in oncology as therapy for many diseases like leukemias and lymphomas, as well as breast, lung, and other solid cancers [1]. However, its clinical use is limited because of its irreversible cardiotoxicity that can lead to heart failure in a dose-dependent manner [2]. Doxorubicin exerts cardiotoxic effects in the heart tissue by enhancing oxidative stress, lipid peroxidation, and inflammation [2, 3]. Several strategies are currently under investigation in preclinical and clinical studies in order to protect the healthy tissues from doxorubicin as well as other anticancer drugs like the anti-Erb2 ones (trastuzumab) [26]. Considering the key role of oxidative stress and inflammation in doxorubicin-induced toxicities, we evaluated the possible use of ozone as a putative cardioprotective strategy in cellular models analyzing possible biological mechanisms involved. The cellular microenvironment plays a crucial role in the pathogenesis of cardiovascular diseases and chemotherapy-related cardiotoxicity. Among the different interleukins and chemokines involved in the pathophysiology of the heart, the interleukin-1*β* plays a major role in the genesis of chronic inflammation. Ozone is an innovative method for decreasing the inflammation status in preclinical and clinical experiences. Our research group recently demonstrated the abilities of ozone in increasing 5-fluorouracile and cisplatin cytotoxicity and decreasing interleukin secretion in human colon cancer cells [14]. Herein, it was demonstrated, for the first time, multiple biological and biochemical properties of ozone in skin fibroblasts and cardiomyocytes during exposure to doxorubicin analyzing its cytoprotective effect (Figure 1) and anti-inflammatory effects (Figures 4–6). Of particular interest is the inhibition observed in p65/NFkB, considering its well known role in the genesis of VEGF, oncogens, and several proinflammatory interleukins involved in skin and cardiovascular damages [27]. The results of the present study demonstrated that the treatments with ozone significantly reduced the production of multiple cytokines involved in cardiotoxicity and skin damages induced by doxorubicin at subclinical concentration. In addition, treatments with ozone significantly increased the cell mitochondrial bioenergetics in skin fibroblasts and cardiomyocytes during incubation with doxorubicin; to preserve the cardiac mitochondrial function during chemotherapy is clinically important because the anthracycline cell damages start from the mitochondria and the enzymes belonging to oxidative phosphorylation. The mitochondrial membrane potential is a useful indirect tool for the evaluation of mitochondria metabolism, and we believe that the results obtained with the use of ozone are of potential biochemical interest for clinicians. Interestingly, the anti-inflammatory and antioxidant effects of ozone are mainly mediated by the activation of Nrf2 (Figure 5) also in cardiomyocytes and skin fibroblasts. These results could be of translational interest in oncology considering that Nrf2 is one of the main therapeutic targets in cardiovascular diseases as well as in cancer [23]. In conclusion, the results of the present study demonstrated, for the first time, that the treatment with ozone has anti-inflammatory and cytoprotective effects on both skin fibroblasts and cardiomyocytes during incubation with doxorubicin at subclinical concentration through three mechanisms: (1) the activation of Nrf2-related functions; (2) the inhibition of leukotriene B4 and p65/NF-kB expressions carrying a general anti-inflammatory effect; and (3) the improvements of mitochondrial potential and consequently its cellular functions. The present study indicates that ozone could be a potentially effective tool for the management of cardiac and skin microenvironments; based on these findings, we are planning preclinical studies of cardioprotection in cancer-bearing mice treated with doxorubicin, in order to analyze the effects of ozone on cardiac parameters like troponin as well as on the global longitudinal strain (GLS) and left ventricular ejection fraction.

## Data Availability

The data used to support the findings of this study are available from the corresponding author upon request.
